# Heterologous Expression and Characterization of a Thermostable α-L-Rhamnosidase from *Thermoclostridium* *stercorarium* subsp. *thermolacticum* DSM 2910 and Its Application in the Biotransformation of Rutin

**DOI:** 10.4014/jmb.2305.05032

**Published:** 2023-07-21

**Authors:** Lin Ge, Yingying Liu, Fangming Zhou, Lingling Zhan, Linguo Zhao

**Affiliations:** 1 Department of Medical Science and Technology, Suzhou Chien-Shiung Institute of Technology, 1 Jian Xiong Road, Taicang 215411, P.R. China; 2 Jiangsu Co-Innovation Center of Efficient Processing and Utilization of Forest Resources, Nanjing Forestry University, Nanjing 210037, P.R. China; 3 College of Chemical Engineering, Nanjing Forestry University, 159 Long Pan Road, Nanjing 210037, P.R. China

**Keywords:** *Thermoclostridium* *stercorarium* subsp. *thermolacticum* DSM 2910, α-L-rhamnosidase, thermal stability, isoquercitrin

## Abstract

An α-L-rhamnosidase gene from *Thermoclostridium*. *stercorarium* subsp. *thermolacticum* DSM 2910 (TstRhaA) was cloned and expressed. The maximum TstRhaA activity of the protein reached 25.2 U/ml, and the molecular mass was approximately 106.6 kDa. The protein was purified 8.0-fold by Ni-TED affinity with an overall recovery of 16.6% and a specific activity of 187.9 U/mg. TstRhaA activity was the highest at 65°C and pH 6.5. In addition, it exhibited excellent thermal stability, better pH stability, good tolerance to low concentrations of organic reagents, and high catalytic activity for *p*-nitrophenyl-α-L-rhamnopyranoside (*p*NPR). Substrate specificity studies showed that TstRhaA exhibited a high specific activity for rutin. At 60°C, pH 6.5, and 0.3 U/ml enzyme dosage, 60 g/l rutin was converted to 45.55 g/l isoquercitrin within 150 min. The molar conversion rate of rutin and the yield of isoquercitrin were 99.8% and 12.22 g/l/h, respectively. The results suggested that TstRhaA could be used for mass production of isoquercitrin.

## Introduction

Isoquercitrin is a rare flavonol glycoside that exhibits anti-cancer, anti-influenza, antioxidant, anti-allergic, antihypertensive and other pharmacological activities [1-5], and is attracting increasing attention. Isoquercitrin is also a key synthetic intermediate for the preparation of enzymatically modified isoquercitrin, which is the only flavonoid food additive approved by the US FDA, and the GRAS Notice for EMIQ is GRN 000220 [6, 7]. However, extracting and isolating isoquercitrin from plants on a large scale is difficult because the content of isoquercitrin in nature is low, greatly restricting its large-scale application [8]. Rutin is abundant in many plants [9], and isoquercitrin differs from rutin by only one rhamnose residue. Therefore, concerting multicomponent rutin into the rare component isoquercitrin is a good strategy [10-13].

α-L-Rhamnosidase (E.C. 3.2.1.40) is ubiquitous in natural sources, such as fungi, mammalian tissues, plants, and bacteria [14-16]. It can efficiently and specifically cleave a variety of natural compounds containing nonreducing terminal L-rhamnose residues, such as hydrolyzed rutin as isoquercitrin and icaritin C as icariin [17, 18]. In addition, a few α-L-rhamnosidases can catalyze the reverse hydrolytic synthesis of rhamnosides using rhamnose as a donor, such as the rhamnosylation of mannitol and the rhamnosylation of phenolic compounds [19, 20]. α-L-rhamnosidase belongs to the GH78, GH13, and GH106 glycoside hydrolase families [21], among which the GH78 family is the majority. This enzyme has many applications in industry [22]. However, most reported α-L-rhamnosidase is not very thermostable at medium and high temperatures, which greatly limits its industrial application.

The CSTERTH_05025 protein of the GH78 family from *Thermoclostridium* *stercorarium* subsp. *thermolacticum* DSM 2910 was listed in the Carbohydrate-Active enZYmes (CAZy) database. We speculated that the enzyme should exhibit α-L-rhamnosidase activity and that the enzyme might be more thermostable. Therefore, in our study, the CSTERTH_05025 protein gene from *Thermoclostridium* *stercorarium* subsp. *thermolacticum* DSM 2910 was cloned, expressed and characterized. The results showed that the enzyme exhibits α-L-rhamnosidase activity and good thermal stability. Moreover, the conditions for the conversion of rutin to isoquercitrin were optimized, and the results showed that the enzyme exhibits high selectivity for the transformation of rutin to isoquercitrin. Our results suggested that TstRhaA shows great potential for hydrolyzing rutin to prepare isoquercitrin.

## Materials and Methods

### Strains, Plasmids, and Materials

*E. coli* DH5α and *E. coli* BL21(DE3) were purchased from TransGen Biotechnology (China). The genomic DNA from *Thermoclostridium* *stercorarium* subsp. *thermolacticum* DSM 2910 was purchased from DSMZ (www.dsmz.de). The expression vector pET-20b (Novagen) was employed as a cloning and expression vector.

The DNA purification kit, Plasmid MiniPrep Kit, DNA Marker, restriction endonucleases NcoI, XhoI, and PCR SuperMix were purchased from TransGen Biotechnology (China). Artificial substrates of the *p*NP series, hesperidin, and naringin were obtained from Sigma‒Aldrich (USA). Rutin and myricetrin were purchased from Chendu Must Bio-Technology (China). The Ni-TED affinity column, T4 DNA ligase, and modified Bradford protein assay kit were obtained from Sangon Biotech (China).

### Plasmid and Recombinant Bacteria Constructions

The TstRhaA gene was amplified from *T. stercorarium* DSM 2910 genomic DNA using PCR SuperMix by PCR. Primers TstRhaA-F (CATG *CCATGG* AAATGATGAGAGTTTA TAAC) and TstRhaA -R (CCG *CTCGAG* TTCCACAGTAACTTTGCTGAG) were used for gene amplification. The underlined bases are restriction enzyme sites. The PCR product was cleaved using the restriction enzymes NcoI and XhoI and then inserted into pET-20b (+) to generate the expression plasmid pET-TstRhaA. Finally, the positive expression plasmid pET-TstRhaA was transformed into *E. coli* BL21(DE3) and then screened by ampicillin resistance.

### Sequence Analysis of TstRhaA

Potential ORFs of TstRhaA were found using the CAZy database. The National Center for Biotechnology Information (NCBI) database was used for BLAST sequence alignment. Several GH78 family α-L-rhamnosidases of different origins were used for sequence alignment. Multiple protein sequence alignment was performed using Clustal X1.9. Molecular weight and theoretical pI were predicted using DNAMAN software.

### Optimization of TstRhaA Culture Conditions

The positive transformants were inoculated in 5 ml LB medium containing ampicillin at a final concentration of 100 mg/l and incubated for 16 h at 37°C and 180 r/min. The cells were then inoculated into 150 ml LB medium containing ampicillin at a final concentration of 100 mg/l according to the 1% inoculum to continue the culture.

By optimizing the concentration of IPTG (0.01, 0.02, 0.05, 0.1, 0.2, 0.5, 1.0 mM), induction temperature (30°C, 33°C, 37°C, 40°C, 42°C), OD_600_ (0.2, 0.4, 0.6, 0.8, 1.0, 1.2), and induction time (4, 6, 8, 10, 12, 14 h), we determined the culture conditions for the highest expression of the enzyme.

### Methods for Purifying TstRhaA

At the end of incubation, the cells were collected by centrifugation, resuspended in 20 mM Tris‒HCl buffer (pH 7.9) containing 5 mM imidazole, and finally crushed by a pressure crusher. The resulting supernatant was purified using a Ni‒TED affinity column, and the purified TstRhaA was eluted with 20 mM Tris-HCl buffer (pH 7.9) containing 1 M imidazole. The expressed proteins were detected by SDS‒PAGE and analyzed by a gel imager. The protein concentration was assayed using a Bradford protein assay kit with bovine serum albumin as the standard [23].

### Methods for Measuring Enzyme Activity

TstRhaA activity assays were performed using *p*NPR as a substrate in 100 μl of reaction solution with 1 mM *p*NPR at 65°C and 100 mM pH 6.5 citrate-phosphate buffer. The reaction was terminated by adding 300 μl of 1 M sodium carbonate after 5 min of reaction. The released p-nitrophenol (*p*NP) was then measured at 405 nm. One unit of TstRhaA activity was defined as the amount of enzyme liberating 1 micromole of *p*NP per minute at 65°C and pH 6.5.

### Determination of Enzymatic Properties

*p*NPR was used to characterize the enzymatic properties of TstRhaA. The optimum pH of TstRhaA was determined by measuring the enzyme activity from pH 5.0 to 8.0. The optimum temperature of TstRhaA was determined by measuring the enzyme activity from 35 to 75°C.

The pH stability of TstRhaA was assessed by measuring the remaining enzyme activity after TstRhaA was incubated for 4 h at 4°C, 45°C, and 60°C in a buffer pH range of 5.0 to 8.0. The thermal stability of TstRhaA was determined by measuring the remaining enzyme activity after TstRhaA was incubated for different times at 60°C and 65°C in pH 6.5 buffer.

The organic solvent tolerance of TstRhaA was determined by adding methanol, ethanol, or DMSO to the reaction mixture at final concentrations of 0%, 10%, 15%, and 20%, respectively.

The substrate specificity of the enzyme was tested by using the synthetic substrates *p*NPR, *p*NPGlu, *p*NPArf, *p*NPArp, *p*NPXyl, *p*NPGal and plant-derived flavonoid compounds rutin, myricetrin, hesperidin, and naringin. The specific enzymatic activity of TstRhaA against different substrates was determined in the reaction system at a substrate concentration of 1.0 mM.

Different *p*NPR concentrations (0.1, 0.2, 0.4, 0.6, 0.8, and 1 mM) were measured at pH 6.5 and 65°C to determine the initial rate TstRhaA kinetic constants.

### Analysis of Rutin Degradation

The reaction system was 200 μl, which contained rutin, buffer, and TstRhaA. The effects of different pH values, temperature, enzyme amounts and reaction times on the conversion rate of hydrolyzed rutin were studied. The hydrolysis of rutin by TstRhaA was investigated by HPLC. The HPLC method was the same as we previously performed [24]. The rutin transformation rate and isoquercitrin generation yield were calculated as follows.

$$\text{Rutin transformation rate=}\left(\text{1-}\frac{\text{molar amount of rutin (mM)}}{\text{initial molar amount of rutin (mM)}}\right)\text{×100\%}$$

$$\text{Isoquercitrin generation yieled=}\frac{\text{molar amount of isoquercitrin (mM)}}{\text{initial molar amount of rutin(mM)}}\text{×100\%}$$

### Statistical Analysis

All experiments were repeated three times. Data represent the means ± SDs of three replicates. Tukey’s test was used at 95% confidence intervals (*p*<0.05).

## Results and Discussion

### Analyzing the Sequence of TstRhaA

**Fig. 1:**
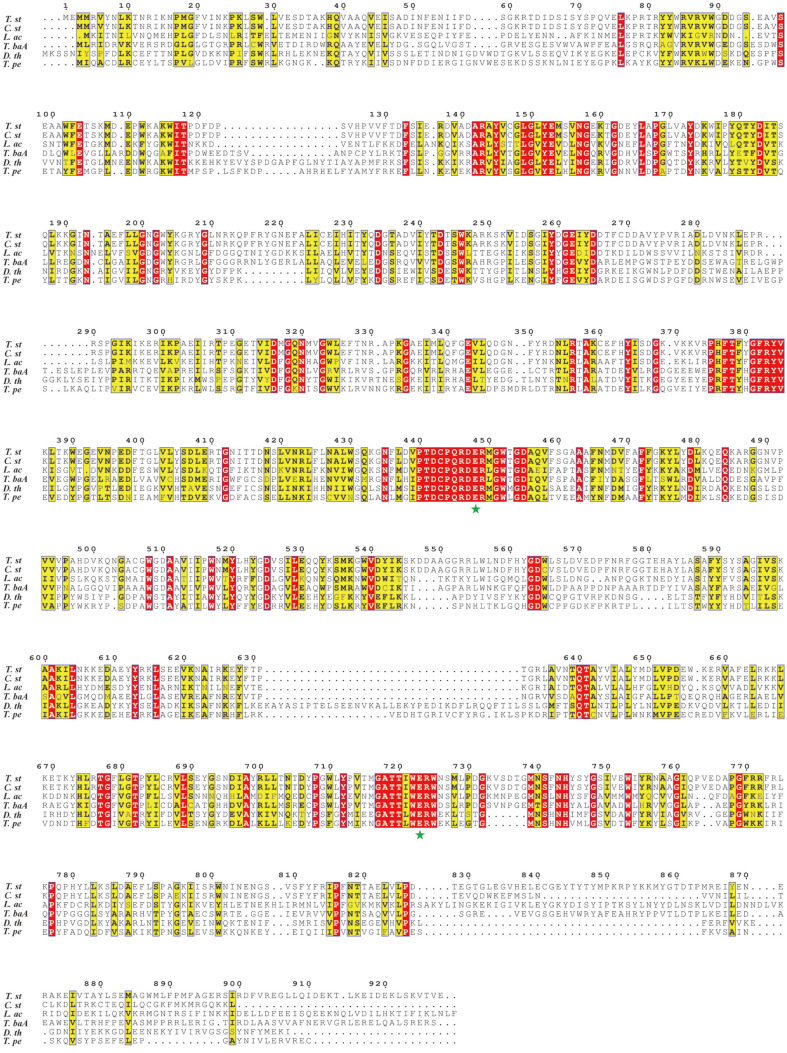
Comparison of the sequences of TstRhaA with family 78 glycoside hydrolases by using multialignment. Multiple sequence alignment of TstRhaA with selected glycoside hydrolase family 78 enzymes was performed by using Clustal X2.0. Full species names and GenBank IDs of the selected glycoside hydrolases in family 78 are as follows: *Thermoclostridium* *stercorarium* subsp. *thermolacticum* DSM 2910 (*T. st*), [GenBank: ANW98449.1]; *Clostridium stercorarium* (*C. st*), [GenBank: CAB53341.1]; *Lactobacillus acidophilus* NCFM (*L. ac*), [GenBank: AAV43293.1]; *Thermomicrobia bacterium* PRI-1686 (*T. baA*), [GenBank: AAR96046.1]; *Dictyoglomus thermophilum* H-6-12 (*D. th*), [GenBank: ACI19983.1]; *Thermotoga petrophila* RKU-1 (*T. pe*), [GenBank: ABQ47687.1]. Strictly conserved residues are highlighted with red shaded boxes, and moderately conserved residues are boxed. The proposed general acid and base are marked with green stars. This figure was generated by ESPRIPT 3.0 [31].

The full-length α-L-rhamnosidase gene TstRhaA (GenBank Accession No. ANW98449.1, CSTERTH_05025) from *Thermoclostridium* *stercorarium* subsp. *thermolacticum* DSM 2910 was 2787 bp in length, encoding 928 amino acids with a pI of 5.5 and a predicted molecular mass of 106.6 kDa. TstRhaA exhibited a high homology with the α-L-rhamnosidase from *Clostridium stercorarium* (89.33% identity, GenBank Accession No. CAB53341.1) and *Clostridiaceae bacterium* (82.24% identity, GenBank Accession No. NLX78030.1). Comparison of the TstRhaA cluster with several bacterial-derived α-L-rhamnosidase of the GH78 family revealed that both possessed the same proposed general acid and base, Glu (Fig. 1) [25], suggesting that TstRhaA belongs to the bacterial members of the GH78 family. Furthermore, several charged residues (Asp442, Arg446, Asp447, Arg449, and Asp455) along the proposed general acid Glu448 were completely conserved and thus formed a conserved amino acid motif PTDCPQRDERMGWTGDA (residues 440-456). Furthermore, charged residues Arg724 along the proposed general base Glu723 were completely conserved and thus formed a conserved amino acid motif GATTIWERW (residues 717-725). The conserved amino acid motifs form the conserved catalytic domain and affect the catalytic activity of the enzyme.

### Optimization of Culture Conditions

**Fig. 2:**
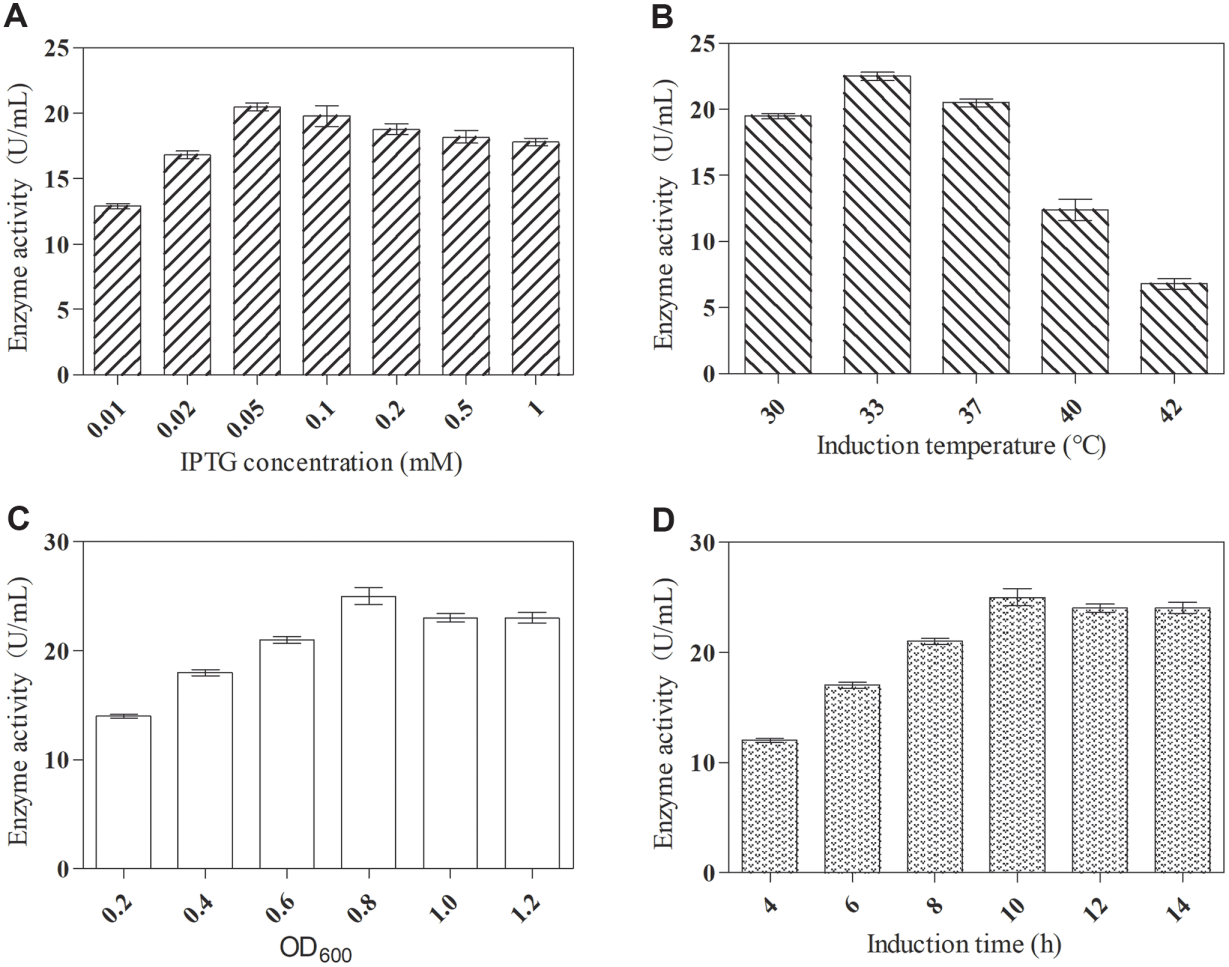
Optimization of culture conditions of TstRhaA. (**A**) Effect of IPTG concentration on enzyme activity; (**B**) Effect of induction temperature on enzyme activity; (**C**) Effect of OD_600_ on enzyme activity; (**D**) Effect of induction time on enzyme activity. Data represent the means of three experiments, and error bars represent the standard deviation.

The culture conditions for the recombinant strain producing the target protein were optimized. As shown in Fig. 2A, the optimal concentration of the inducer was 0.05 mM, which indicated that 0.05 mM IPTG was the optimal concentration to induce transcription of the target gene. The optimal induction temperature was 33°C (Fig. 2B), which showed that the speed of protein synthesis and rate by which intermediates fold into aggregates were optimal at 33°C. Moreover, the optimal OD_600_ was 0.8 (Fig. 2C), indicating that the maximum balance between bacterial growth and protein expression could be achieved under this condition. Finally, the maximum enzyme activity was observed at 10 h after induction (Fig. 2D). The possible reason was that the enzyme began to be degraded by proteases after 10 h induction. Under optimal culture conditions, the target protein TstRhaA exhibited high α-L-rhamnosidase activity with approximately 25.2 U/ml, indicating that TstRhaA was highly expressed.

### Purification of Recombinant TstRhaA

**Fig. 3:**
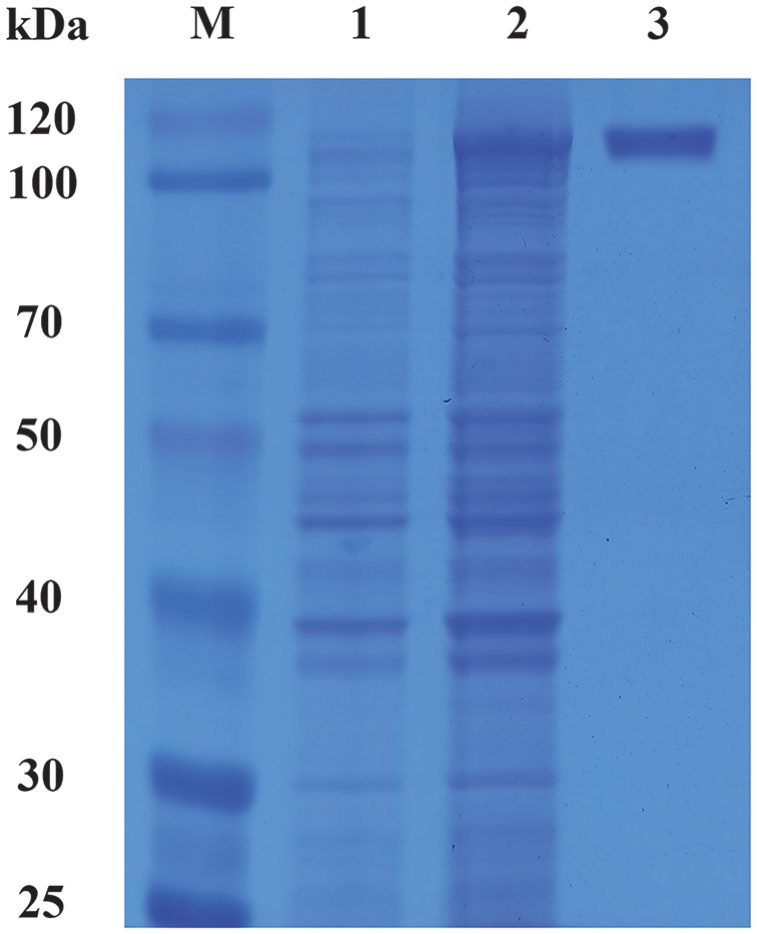
SDS‒PAGE analysis of TstRhaA expressed in *E. coli* BL21 (DE3). Lane M: protein molecular mass marker, Lane 1: the crude extract of *E. coli* BL21 (DE3) harboring pET-20b (+), Lane 2: the crude extracts of *E. coli* BL21 (DE3) harboring pET-TstRhaA, Lane 3: TstRhaA purified by Ni–TED resin affinity chromatography.

A Ni-TED-affinity column was used to purify TstRhaA. Finally, the yield of TstRhaA reached 16.6%, and the specific activity of purified TstRhaA was 187.9 U/mg, which was 8.0-fold higher than that of the crude enzyme (Table 1). The final obtained TstRhaA showed only one band on the SDS‒PAGE gel, and its relative molecular mass was approximately 106.6 kDa, as shown by the results (Fig. 3, Lane 3).

### Characterization of Recombinant TstRhaA

**Fig. 4:**
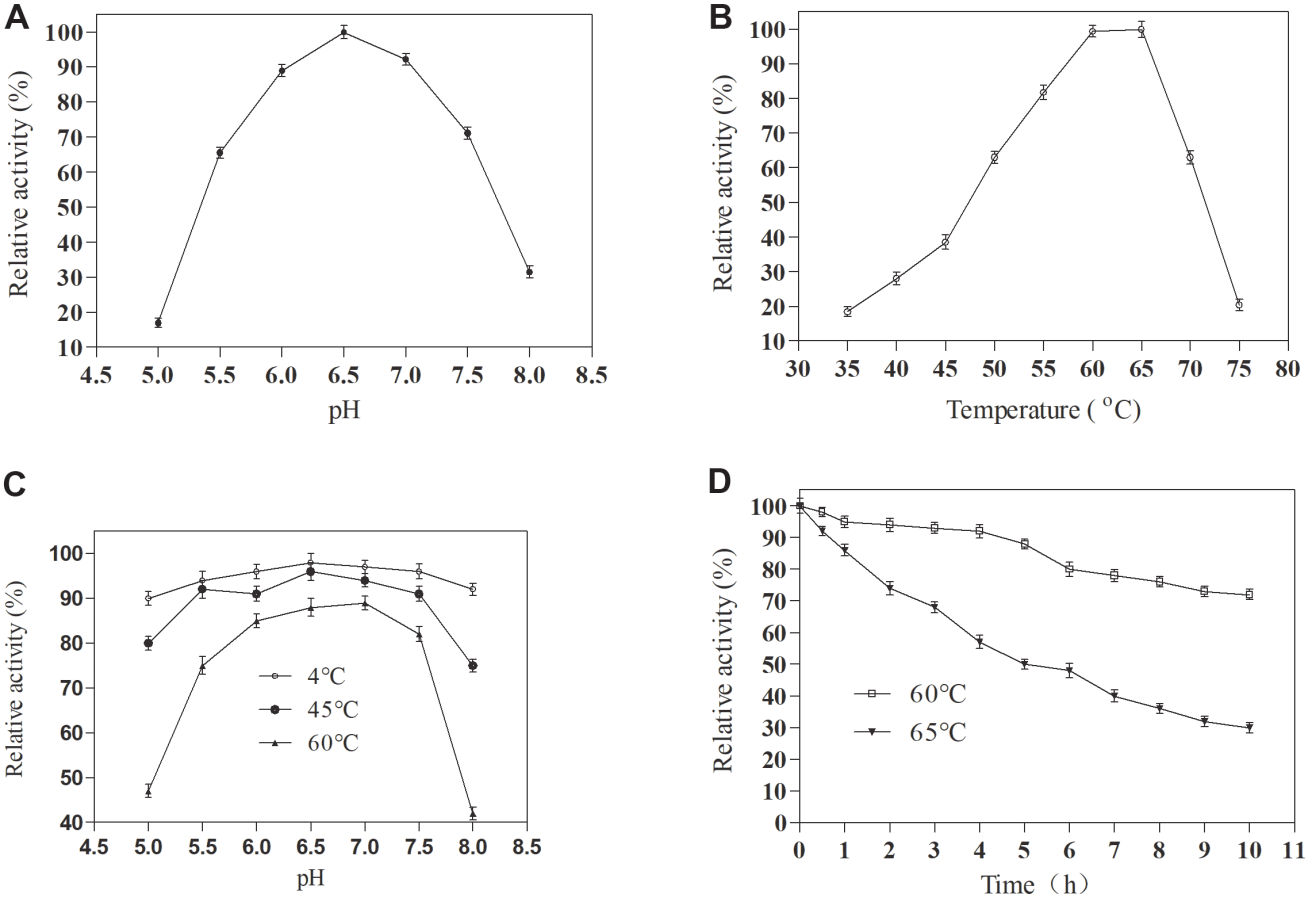
The effects of pH and temperature on the activity and stability of TstRhaA. (**A**) Effect of pH on TstRhaA activity. (**B**) Effect of temperature on TstRhaA activity. (**C**) The pH stability of the enzyme TstRhaA. (**D**) The thermostability of the enzyme TstRhaA; the residual activity was monitored, while the enzyme was incubated at 60°C (filled circles) and 65°C (filled inverted triangles). The initial activity was defined as 100%. These activities were expressed as relative values. Data represent the means of three experiments, and error bars represent the standard deviation.

The enzyme properties of TstRhaA were investigated using *p*NPR as a substrate. As shown in Fig. 4A, the enzyme activity of TsRhaA was maximal at pH 6.5, and the enzyme activity was more than 65% of the maximum activity in a pH range of 5.5 to 7.5. The enzyme activity of TstRhaA was the highest at 65°C (Fig. 4B), and the results showed that this enzyme was a medium- and high-temperature enzyme, similar to α-L- rhamnocidase derived from *Aspergillus niger* [26], *Aspergillus oryzae* NL-1 [19], *Aspergillus terreus* CCF 3059 [27] and *Thermophilic* strain PRI-1686 [28] (Table 2). The pH stability of TstRhaA was further determined. The results showed that more than 94%, 90%, and 75% of the original enzyme was maintained by the residual enzyme activity of purified TstRhaA after incubation at 4°C, 45°C, and 60°C for 4 h in a pH range of 5.5 to 7.5 (Fig. 4C), respectively. However, the residual enzyme activity of α-L-rhamnosidase from *Paenibacillus odorifer* only retained more than 85% of the original enzyme activity after incubation at 4°C and 45°C for 1 h in the pH range of 5.5 to 7.5 [29]. In addition, the residual enzyme activity of α-L-rhamnosidase from *Bacteroides thetaiotaomicron* also only retained more than 60% of the original enzyme activity after incubation at 45°C for 4 h in the pH range of 5.5 to 7.5 [18]. Therefore, the TstRhaA has better pH stability and is suitable for use under neutral conditions. In industrial applications, the thermal stability of enzymes is a very important parameter. Therefore, the thermal stability of TstRhaA was determined at 60°C and 65°C (Fig. 4D). TstRhaA was highly stable at 60°C and 65°C, at which it maintained more than 70% of the initial activity at 10 h and more than 50% of the initial activity at 5 h. However, the residual activity of two α-L-rhamnosidase from *Thermophilic bacterium* PRI-1686, RhmA and RhmB, was lower than 40% and 60% after 8 h incubation at 60°C, respectively. Moreover, the half-life of the α-L-rhamnosidase from *A. terreus* CCF 3059 was only 127.9 min at 65°C. The residual activity of α-L-rhamnosidase from *A. niger* retained at 60% after 1 h of incubation at 65°C. The results showed that higher temperatures are better when using TstRhaA, which can reduce the viscosity of the substrate, improve the solubility of the substrate, and reduce the risk of microbial contamination [19], which is beneficial for industrial applications.

**Fig. 5:**
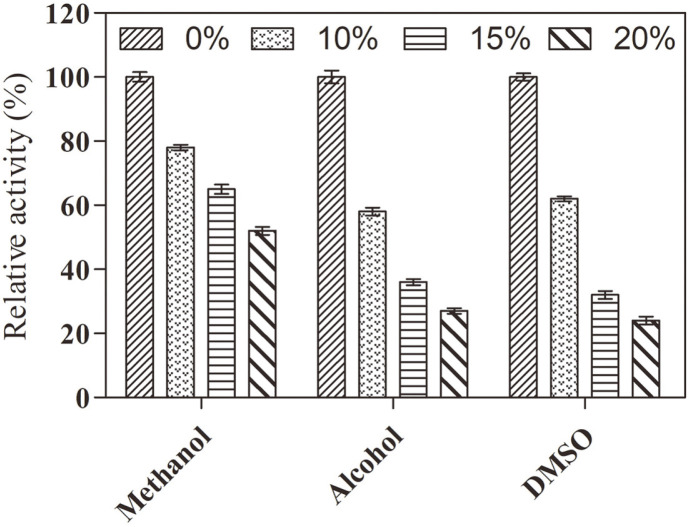
The effect of organic solvents, including methanol, alcohol, and DMSO, on TstRhaA activity. The final concentration of substrate was 1.0 mM. Double distilled water was used instead of the organic solvent for the control. The average activity of the control from three experiments was defined as 100%. Others were expressed as residual activity values. Data represent the means of three experiments, and error bars represent the standard deviation.

For poorly water-soluble substrates, some cosolvents are generally added to the enzymatic conversion system, such as methanol, alcohol, and dimethyl sulfoxide (DMSO). Therefore, we determined the effect of organic solvents on TstRhaA activity. As shown in Fig. 5, TstRhaA retained 50% of its remaining enzyme activity at methanol concentrations below 20%. However, the enzyme activity of TstRhaA decreased significantly when the alcohol and DMSO concentrations exceeded 15%, and the residual enzyme activity was less than 40%. The inhibition of TstRhaA by the three organic reagents was as follows: methanol > DMSO > alcohol, which was consistent with α-L-rhamnosidase from *Aspergillus oryzae* and *Novosphingobium* sp. PP1Y [19, 30]. These results suggest that TstRhaA can be used in reaction systems containing lower concentrations of organic solvents.

### Substrate Specificity and Enzyme Kinetic Assays

A range of *p*-nitrophenyl-based artificial substrates were selected to test the substrate specificity of TstRhaA. TstRhaA was active against *p*NPR but not against *p*NPGlu, *p*NPArf, *p*NPArp, *p*NPGal, or *p*NPXyl. These results indicated that TstRhaA is active only against α-L-rhamnosidase. Additionally, the substrate specificity of TstRhaA was determined for four plant-derived flavonoids, including myricetrin, naringin, hesperidin, and rutin (Table 3). The results showed that TstRhaA can hydrolyze natural products containing α-1,2 and α-1,6 glycosidic bonds but not α-1 glycosidic bond natural products, which is consistent with α-L-rhamnosidase from *A. oryzae* [19].

Michaelis‒Menten parameters were determined by enzyme kinetics studies using *p*NPR as a substrate at optimal temperature and pH. Table 2 shows that the *K_m_*, *V_max_*, *kcat*, and k *kcat*/*K_M_* of the enzyme were 0.36 mM, 368.3 U/mg, 650 s^-1^, and 1,810 s^-1^mM^-1^, respectively, when *p*NPR was used as a substrate. The *V_max_* values were higher than those of α-L-rhamnosidase from *A. oryzae*, *A. terreus*, *Thermophilic bacterium* PRI-1686, and A. niger [19, 26-28]. Furthermore, the lower *K_m_* value and higher *kcat*/*K_m_* values also indicate that TstRhaA exhibits high substrate affinity and catalytic activity against *p*NPR among the thermostable α-L-rhamnosidase. Thus, the catalytic kinetics of TstRhaA on *p*NPR also demonstrate that TstRhaA exhibits properties distinct from other thermostable α-L-rhamnosidase.

### Analysis of Rutin Degradation

**Fig. 6:**
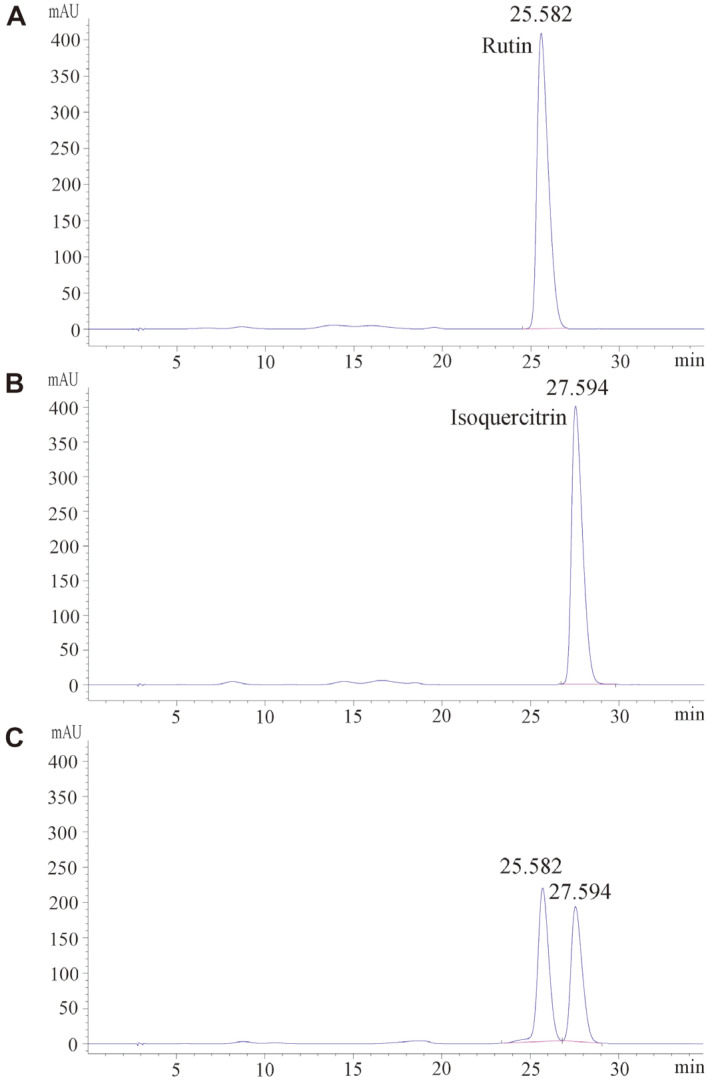
HPLC analysis of rutin hydrolysis by TstRhaA. (**A**) rutin, (**B**) isoquercitrin, (**C**) rutin (60 g/l) incubated with TstRhaA (0.05 U/ml) for 180 min. Data represent the means of three experiments, and error bars represent the standard deviation.

**Fig. 7:**
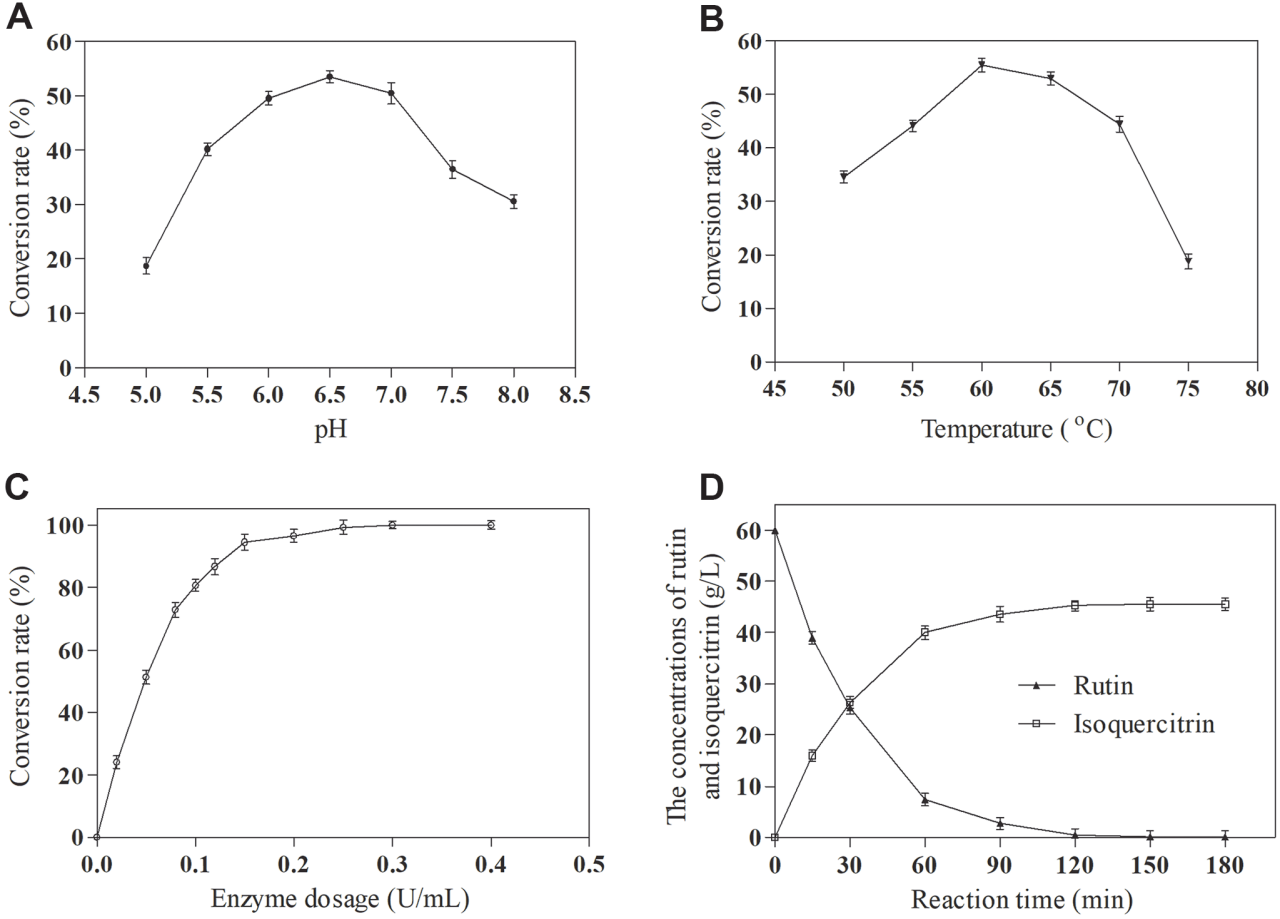
Optimizing the reaction conditions for the conversion of rutin into isoquercetrin by TstRhaA. (**A**) Effect of pH on the transformation rate of rutin; (**B**) Effect of temperature on the transformation rate of rutin; (**C**) Effect of enzyme dosage on the transformation rate of rutin; (**D**) Effect of reaction time on the concentrations of rutin and soquercetrin in the reaction system. Data represent the means of three experiments, and error bars represent the standard deviation.

To verify that rutin undergoes biotransformation by TstRhaA, the hydrolysate was studied by HPLC. As shown in Fig. 6, the final product was identified as isoquercitrin. Furthermore, to determine the optimal conditions for the enzymatic bioconversion of rutin by TstRhaA, the conversion for isoquercitrin preparations was examined at the same substrate concentration and different temperatures, pH conditions, and enzyme dosages. As shown in Fig. 7A, the optimum temperature for hydrolyzing rutin to isoquercitrin was observed at 60°C. This was not consistent with the results obtained by the enzymatic properties measured with *p*NPR as a substrate, which may result from the thermal stability being better at 60°C than at 65°C. Moreover, the optimal pH was revealed to be 6.5 (Fig. 7B), which was identical to the result obtained by the enzymatic properties measured with *p*NPR as the substrate. Subsequently, the optimal enzyme dosage used to convert rutin to isoquercitrin was determined. The results showed that almost all rutin was converted to isoquercitrin in the presence of 0.3 U/ml TstRhaA in a 200 μl reaction system (Fig. 7C). To clarify the course of changes in the concentration of the two components during the reaction, a time-course experiment was carried out, and the hydrolysate was analyzed by HPLC. As shown in Fig. 7D, 60 g/l rutin was converted to 45.55 g/l isoquercitrin after 150 min of reaction. The molar conversion rate of rutin and the yield of isoquercitrin were 99.8% and 12.22 g/l/h, respectively. Compared with α-L-rhamnosidase from other sources (Table 4), TstRhaA is superior in converting high concentrations of rutin to isoquercetin.

In this work, a novel α-L-rhamnosidase from *Thermoclostridium* *stercorarium* subsp. *thermolacticum* DSM 2910 was overexpressed and characterized. TstRhaA displayed higher optimal temperature, better thermal stability, and good tolerance to organic reagents, which is beneficial for application in industry. Furthermore, the enzyme exhibited high catalytic activity for the conversion of rutin to isoquercetin. Therefore, this study presents a novel α-L-rhamnosidase that could be used for the mass production of isoquercitrin.

## Figures and Tables

**Table 1:** Purification scheme for the recombinant protein TstRhaA.

Purification step	Total protein (mg)	Total activity (U)	Specific activity (U/mg)	Yield (%)	Purification factor (fold)
Crude extract	114.8	2709.3	23.6	100.0	1.0
Ni–TED resin affinity chromatography	2.4	450.9	187.9	16.6	8.0

**Table 2:** Enzymatic properties of thermostable α-L-rhamnosidase.

Strain	Temperature* (°C)	*K_M_* (mM)	*Vmax* (U/mg)	*k_cat_* (s^-1^)	*k_cat_*/*K_M_* (s^-1^ mM^-1^)	Reference
*Thermoclostridium* *stercorarium* subsp. *thermolacticum* DSM 2910	65	0.36	368.3	650	1810	This study
*Aspergillus oryzae* NL-1	65	5.2	ND ^#^	1624	312	[19]
*Aspergillus terreus*	65	0.476	ND ^#^	412	867	[27]
*Thermophilic bacterium* PRI-1686 (RhmA)	70	0.46	134	460	1000	[28]
*Thermophilic bacterium* PRI-1686 (RhmB)	70	0.66	356	1254	1900	
*Aspergillus niger*	65	2.9	20.6	ND ^#^	ND ^#^	[26]

*The temperature indicates optimal temperature.

^#^Not detected, the value was not detected based on the references.

**Table 3:** Natural substrate specificity of TstRhaA.

Substrate ^a^	Glycosidic bonds	Relative enzyme activity (Mean% ± SD)
Rutin	α-1,6	100 ± 0.0^b^
Hesperidin	α-1,6	87.6 ± 1.1
Naringin	α-1,2	31.0 ± 0.6
Myricetrin	α-1	ND^c^

^a^Substrate concentration 1.0 mM.

^b^The activity against rutin was assumed to be 100%.

^c^Not detected, relative enzyme activity is not detected.

**Table 4:** Different sources of α-L-rhamnosidase in the conversion of rutin to isoquercitrin.

Strain	Rutin concentration (g/l)	Molar conversion rate (%)	Isoquercitrin productivity (g/l/h)	References
*T. stercorarium* subsp. *thermolacticum* DSM 2910	60	99.8	12.22	This study
*A. niger* CCTCC M 2018240	20	91.42	12.78	[10]
*A. niger* JMU-TS528	1	96.44	0.73	[11]
*C. aurantiacus*	18.32	100	6.97	[12]
*B. breve*	12.21	91.25	8.47	[13]

## References

[1] Cho WK, Lee MM, Ma JY (2022). Antiviral effect of isoquercitrin against influenza A viral infection via modulating hemagglutinin and neuraminidase. Int. J. Mol. Sci..

[2] Kolesarova A, Michalcova K, Roychoudhury S, Baldovska S, Tvrda E, Vasicek J (2021). Antioxidative effect of dietary flavonoid isoquercitrin on human ovarian granulosa cells HGL5 in vitro. Physiol. Res..

[3] Park J (2020). Anti-anaphylactic activity of isoquercitrin (Quercetin-3-O-beta-d-Glucose) in the cardiovascular system of animals. Biomedicines.

[4] Wu P, Liu S, Su J, Chen J, Li L, Zhang R (2017). Apoptosis triggered by isoquercitrin in bladder cancer cells by activating the AMPK-activated protein kinase pathway. Food Funct..

[5] Gasparotto Junior A, Gasparotto FM, Lourenco EL, Crestani S, Stefanello ME, Salvador MJ (2011). Antihypertensive effects of isoquercitrin and extracts from *Tropaeolum majus* L.:evidence for the inhibition of angiotensin converting enzyme. J. Ethnopharmacol..

[6] Wang J, Sun GX, Yu L, Wu FA, Guo XJ (2013). Enhancement of the selective enzymatic biotransformation of rutin to isoquercitrin using an ionic liquid as a co-solvent. Bioresour. Technol..

[7] Shimada Y, Dewa Y, Ichimura R, Suzuki T, Mizukami S, Hayashi SM (2010). Antioxidant enzymatically modified isoquercitrin suppresses the development of liver preneoplastic lesions in rats induced by beta-naphthoflavone. Toxicology.

[8] Gasparotto Junior A, Prando TB, Leme Tdos S, Gasparotto FM, Lourenco EL, Rattmann YD (2012). Mechanisms underlying the diuretic effects of *Tropaeolum majus* L.extracts and its main component isoquercitrin. J. Ethnopharmacol..

[9] Jiang P, Burczynski F, Campbell C, Pierce G, Austria JA, Briggs CJ (2007). Rutin and flavonoid contents in three buckwheat species *Fagopyrum esculentum*, *F. tataricum*, and *F. homotropicum* and their protective effects against lipid peroxidation. Food Res. Int..

[10] Wang D, Zheng P, Chen P, Wu D (2021). Immobilization of alpha-L-rhamnosidase on a magnetic metal-organic framework to effectively improve its reusability in the hydrolysis of rutin. Bioresour. Technol..

[11] Li LJ, Liu XQ, Du XP, Wu L, Jiang ZD, Ni H (2020). Preparation of isoquercitrin by biotransformation of rutin using α-Lrhamnosidase from *Aspergillus niger* JMU-TS528 and HSCCC purification. Prep. Biochem. Biotechnol..

[12] Shin KC, Seo MJ, Oh DK, Choi MN, Kim DW, Kim YS (2019). Cloning and characterization of alpha-L-rhamnosidase from *Chloroflexus aurantiacus* and its application in the production of isoquercitrin from rutin. Biotechnol. Lett..

[13] Zhang R, Zhang BL, Xie T, Li GC, Tuo Y, Xiang YT (2015). Biotransformation of rutin to isoquercitrin using recombinant α-Lrhamnosidase from *Bifidobacterium breve*. Biotechnol. Lett..

[14] Lou H, Liu X, Liu S, Chen Q (2022). Purification and characterization of a novel alpha-L-rhamnosidase from *Papiliotrema laurentii* ZJU-L07 and its application in production of icariin from epimedin C. J. Fungi.

[15] Ferreira-Lazarte A, Plaza-Vinuesa L, de Las Rivas B, Villamiel M, Munoz R, Moreno FJ (2021). Production of α-rhamnosidases from *Lactobacillus plantarum* WCFS1 and their role in deglycosylation of dietary flavonoids naringin and rutin. Int. J. Biol. Macromol..

[16] Singh P, Sahota PP, Singh RK (2015). Evaluation and characterization of new α-L-rhamnosidase-producing yeast strains. J. Gen. Appl. Microbiol..

[17] Yadav V, Yadav PK, Yadav S, Yadav KDS (2010). α-L-rhamnosidase: a review. Process Biochem..

[18] Wu T, Pei J, Ge L, Wang Z, Ding G, Xiao W (2018). Characterization of a α-l-rhamnosidase from *Bacteroides thetaiotaomicron* with high catalytic efficiency of epimedin C. Bioorg. Chem..

[19] Ge L, Xie J, Wu T, Zhang S, Zhao L, Ding G (2017). Purification and characterisation of a novel α-L-rhamnosidase exhibiting transglycosylating activity from *Aspergillus oryzae*. Int. J. Food Sci. Technol..

[20] De Winter K, Šimčíková D, Schalck B, Weignerová L, Pelantova H, Soetaert W (2013). Chemoenzymatic synthesis of α-lrhamnosides using recombinant α-l-rhamnosidase from *Aspergillus terreus*. Bioresour. Technol..

[21] Cantarel BL, Coutinho PM, Rancurel C, Bernard T, Lombard V, Henrissat B (2009). The carbohydrate-active enzymes database (CAZy): an expert resource for glycogenomics. Nucleic Acids Res..

[22] de Araújo MEMB, Moreira Franco YE, Alberto TG, Sobreiro MA, Conrado MA, Priolli DG (2013). Enzymatic de-glycosylation of rutin improves its antioxidant and antiproliferative activities. Food Chem..

[23] Bradford MM (1976). A rapid and sensitive method for the quantitation of microgram quantities of protein utilizing the principle of protein-dye binding. Anal. Biochem..

[24] Ge L, Chen A, Pei J, Zhao L, Fang X, Ding G (2017). Enhancing the thermostability of α-L-rhamnosidase from *Aspergillus terreus* and the enzymatic conversion of rutin to isoquercitrin by adding sorbitol. BMC Biotechnol..

[25] Li B, Ji Y, Li Y, Ding G (2018). Characterization of a glycoside hydrolase family 78 alpha-l-rhamnosidase from *Bacteroides thetaiotaomicron* VPI-5482 and identification of functional residues. 3 Biotech.

[26] Manzanares P, de Graaff LH, Visser J (1997). Purification and characterization of an α-L-rhamnosidase from *Aspergillus niger*. FEMS Microbiol. Lett..

[27] Ge L, Li D, Wu T, Zhao L, Ding G, Wang Z (2018). B-factor-saturation mutagenesis as a strategy to increase the thermostability of alpha-L-rhamnosidase from *Aspergillus terreus*. J. Biotechnol..

[28] Birgisson H, Hreggvidsson GO, Fridjónsson OH, Mort A, Kristjánsson JK, Mattiasson B (2004). Two new thermostable α-Lrhamnosidases from a novel thermophilic bacterium. Enzyme Microb. Technol..

[29] Xie J, Zhao J, Zhang N, Xu H, Yang J, Ye J (2022). Efficient production of isoquercitin, icariin and icariside II by a novel thermostable alpha-l-rhamnosidase PodoRha from *Paenibacillus odorifer* with high alpha-1, 6-/alpha-1, 2- glycoside specificity. Enzyme Microb. Technol..

[30] De Lise F, Mensitieri F, Tarallo V, Ventimiglia N, Vinciguerra R, Tramice A (2016). RHA-P: Isolation, expression and characterization of a bacterial α- l -rhamnosidase from *Novosphingobium* sp. PP1Y. J. Mol. Catal. B: Enzymatic..

[31] Robert X, Gouet P (2014). Deciphering key features in protein structures with the new ENDscript server. Nucleic Acids Res..

